# Comparison of young femoral neck fractures treated by femoral neck system, multiple cancellous screws and dynamic hip screws: a retrospectively comparison study

**DOI:** 10.1186/s12891-024-07319-y

**Published:** 2024-03-02

**Authors:** Leyi Cai, Wenhao Zheng, Chunhui Chen, Wei Hu, Hua Chen, Te Wang

**Affiliations:** Department of Orthopaedic Surgery, The Second Affiliated Hospital and Yuying Children’s Hospital of Wenzhou Medical University, 109 Xueyuanxi Road, Wenzhou, Zhejiang 325000 China

**Keywords:** Femoral neck system, Multiple cancellous screws, Dynamic hip screws, Young femoral neck fractures

## Abstract

**Background:**

Implant choice for the fixation of femoral neck fracture is one of the most important management controversies. This study aims to evaluate and compare the short-term outcomes associated with the use of the Femoral Neck System (FNS), Multiple Cancellous Screws (MCS), and Dynamic Hip Screws (DHS) in treating femoral neck fractures in a young patient population.

**Methods:**

From June 2018 to June 2021, a total of 120 surgeries for a primary femoral neck fracture were retrospectively analyzed. This review encompassed demographic details of the patients and the mechanisms behind the injuries. Key surgical parameters such as operation duration, intraoperative blood loss, fluoroscopy duration, and hospital stay were meticulously documented. The employed surgical technique was described. All patients were followed up at 6 weeks, 3 months, 6 months, and 12 months postoperatively. Avascular necrosis of the femoral head (AVN), nonunion, malreduction, implant failure or other complications were noted. The functional status at the last follow-up was assessed using the Harris functional scoring criteria.

**Results:**

There were 90 males and 30 females, with a mean age of 40.4 years. As to patient characteristics, there were no significant differences between the three groups. DHS group showed longer operation time(52.15 ± 4.80 min), more blood loss(59.05 ± 5.87 ml) and longer time of hospitalization(7.6 ± 0.90 d) than FNS group (39.65 ± 2.84 min, 45.33 ± 9.63 ml and 4.87 ± 0.48 d) and MCS group (39.45 ± 3.10 min, 48.15 ± 7.88 ml and 5.04 ± 0.49 d) (*p* < 0.05). In addition, the time of fluoroscopy in FNS group (15.45 ± 3.67) was less than that in MCS group (26.3 ± 4.76) and DHS group (27.1 ± 5.67) (*p* < 0.05). The cost of FNS group(44.51 ± 2.99 thousand RMB) was significantly higher than the MCS and DHS groups. The FNS, MCS and DHS groups showed a similar mean length of femoral neck shortening (LFNS) and Harris score. The FNS, MCS and DHS groups showed a similar mean rate of AVN and internal fixation failure.

**Conclusions:**

Following successful fracture reduction, FNS, MCS, and DHS are effective for in the young femoral neck fractures. No difference was found in complications between the three groups. However, the reduced fluoroscopy time associated with FNS contributes to shorter operation durations. The adoption of minimally invasive techniques correlates with decreased blood loss and shorter hospital stays. Nevertheless, these advantages may be offset by the potential economic burden they impose.

## Background

Globally, hip fractures afflict 4.5 million patients annually, with a substantial portion of these fractures occurring in the femoral neck [1]. This is accompanied by an enormous socioeconomic burden and medical challenge. For old patients (age > 65 years) with femoral neck fractures, hemiarthroplasty and total hip arthroplasty are the common treatment methods for patients with poor physical condition [2], while reduction and internal fixation can serve as an effective treatment option for patients in good physical condition as well. For young patients, osteosynthesis of a femoral neck fracture with close reduction and implants is a relatively smaller surgical procedure with shorter duration of surgery, reduced bleeding and need for blood transfusions, smaller risk of deep wound infections, and reduced length of hospitalization compared with arthroplasty [3]. However, the rate of reoperation following osteosynthesis has been reported to be considerably higher than that following arthroplasty, with mechanical failure as the most common cause [4, 5].

Implant choice for the fixation of femoral neck fracture is one of the most important management controversies in the treatment of these challenging fractures [6, 7]. These fractures are commonly stabilized using various implants such as Multiple Cannulated Screws (MCS), Dynamic Hip Screws (DHS) with or without an antirotation screw, DHS with a blade in place of a screw (DHS-blade), or other comparable devices. Currently, there are no concrete recommendations for treatment of femoral neck fractures. A recent survey of orthopedic surgeons demonstrated a near even split between DHS or MCS when treating displaced femoral neck fractures [8]. Additionally, another quarter of the surveyed surgeons changed their desired implant when in the Operating Room (OR) [9].

**Fig. 1:**
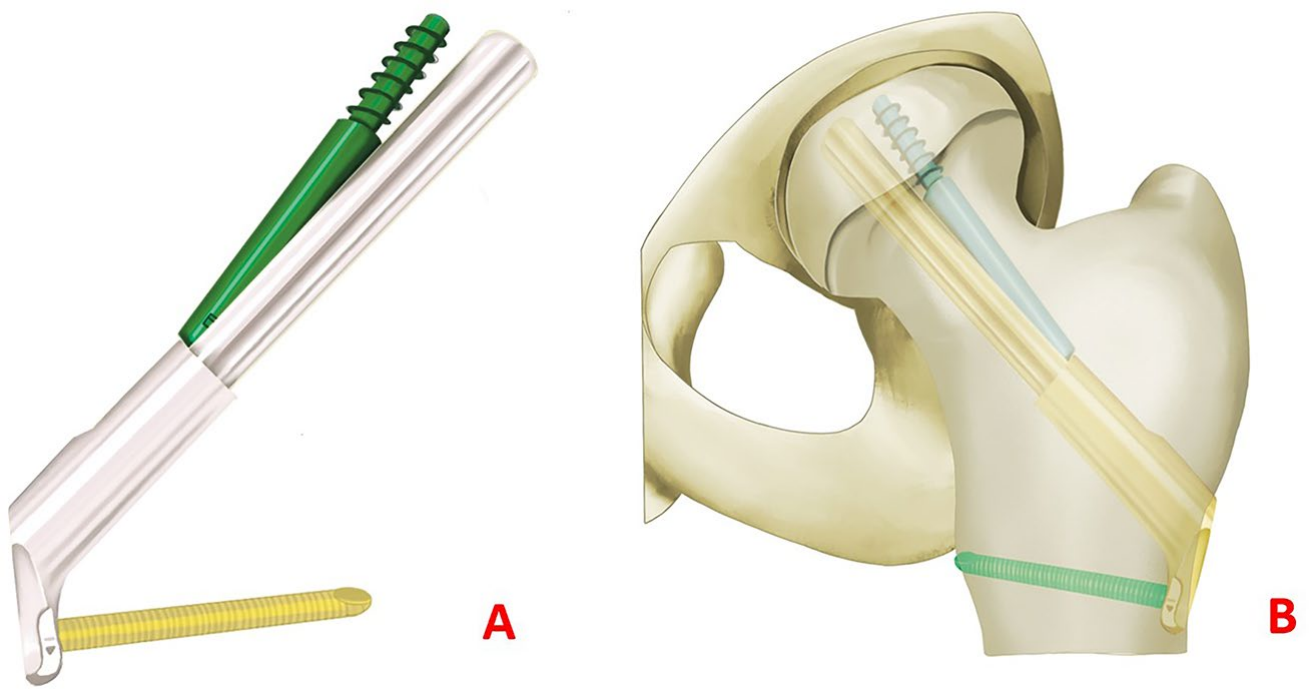
(**A**): Schematic diagram of FNS structure. (**B)**: Schematic diagram of FNS after placement of femoral neck

The role of an ideal minimally invasive implant would be ensuring the required stability of fixation, without pronounced femoral neck shortening or femoral head tilting and rotation. The new minimally invasive implant femoral neck system (FNS, Fig. 1) [10], developed for dynamic fixation of femoral neck fractures, combines the advantages of angular stability with a minimally invasive surgical technique. The primary objective of this study is to assess and compare the short-term outcomes of employing the FNS, MCS, and DHS in treating femoral neck fractures among young patients.

## Methods

### Patients

From June 2018 to June 2021, a total of 120 surgeries for a primary femoral neck fracture (OTA/AO classification, 31-B) were retrospectively analyzed in the Orthopedics Department in our hospital. An informed consent was attested for all the study participations. The inclusion criteria are as follows: (1) diagnosed with femoral neck fracture fromJune 2018 to June 2021, (2) age ≤ 65 years old, (3) undergoing closed reduction and internal fixation surgery with FNS or MCS or DHS, (4) the reduction was of high quality (according to Garden alignment index) [11], and (5) no decreased mobility and other severe hip diseases before femoral neck fracture. The exclusion criteria are as follows: (1) patients with pathological fractures, (2) can’t follow up on time, (3) patients with cognitive dysfunction or mental disorders, (4) patients with acetabular fractures. Ultimately, a total of 120 patients met the criteria and were included in the study.

This research was conducted as a retrospective cohort study, with clinical data sourced from the hospital’s clinical database. Demographic data of patient and mechanism of injury were recorded. Collected data included age, sex, BMI, cause of injury, side of injured, smoking, time to surgery, Garden fracture classification(non-displaced or displaced), Pauwels classification and American Society of Anesthesiologists (ASA) score [12]. Time to surgery was defined as the time from fracture diagnosis (preoperative radiograph) until the onset of surgery. The FNS is from DePuy Synthes, Zuchwil, Switzerland. Additionally, surgical details such as the duration of the operation, intraoperative blood loss, fluoroscopy time, length of hospital stay, and the cost of hospitalization (RMB, thousands) were meticulously documented.

### Surgical methods

Patients were treated with spinal or general anesthesia and were positioned supine on the fracture table. The procedure involved using a fracture table for closed reduction under the guidance of G-arm fluoroscopy. The quality of the fracture reduction was evaluated based on the Garden Index.

For young femoral neck fractures, the primary goal for doctors is to achieve the most precise reduction possible and ensure the stability of the reduction throughout the patient’s full recovery [13]. After successful closed reduction, patients underwent routine surgical procedures for implantation of FNS, DHS, and MCS according to the surgical technique recommended by the manufacturer. In the MCS group, a consistent approach was employed, involving the placement of three or four cancellous screws. These screws were arranged either in an inverted triangle pattern or a quadrilateral pattern, ensuring a parallel trajectory for each screw. In the DHS group, a guide k-wire was inserted into the femoral neck with a 135 angle guide. The length of the DHS-blade was determined shorter than the guide k-wire. The tip of the blade was positioned about 5–10 mm beneath the surface of the femoral cartilage. The side plates were placed closely to the bone surface and fixed with two locking screws [14]. It can be implanted with or without an additional cannulated antirotational screw.

**Fig. 2:**
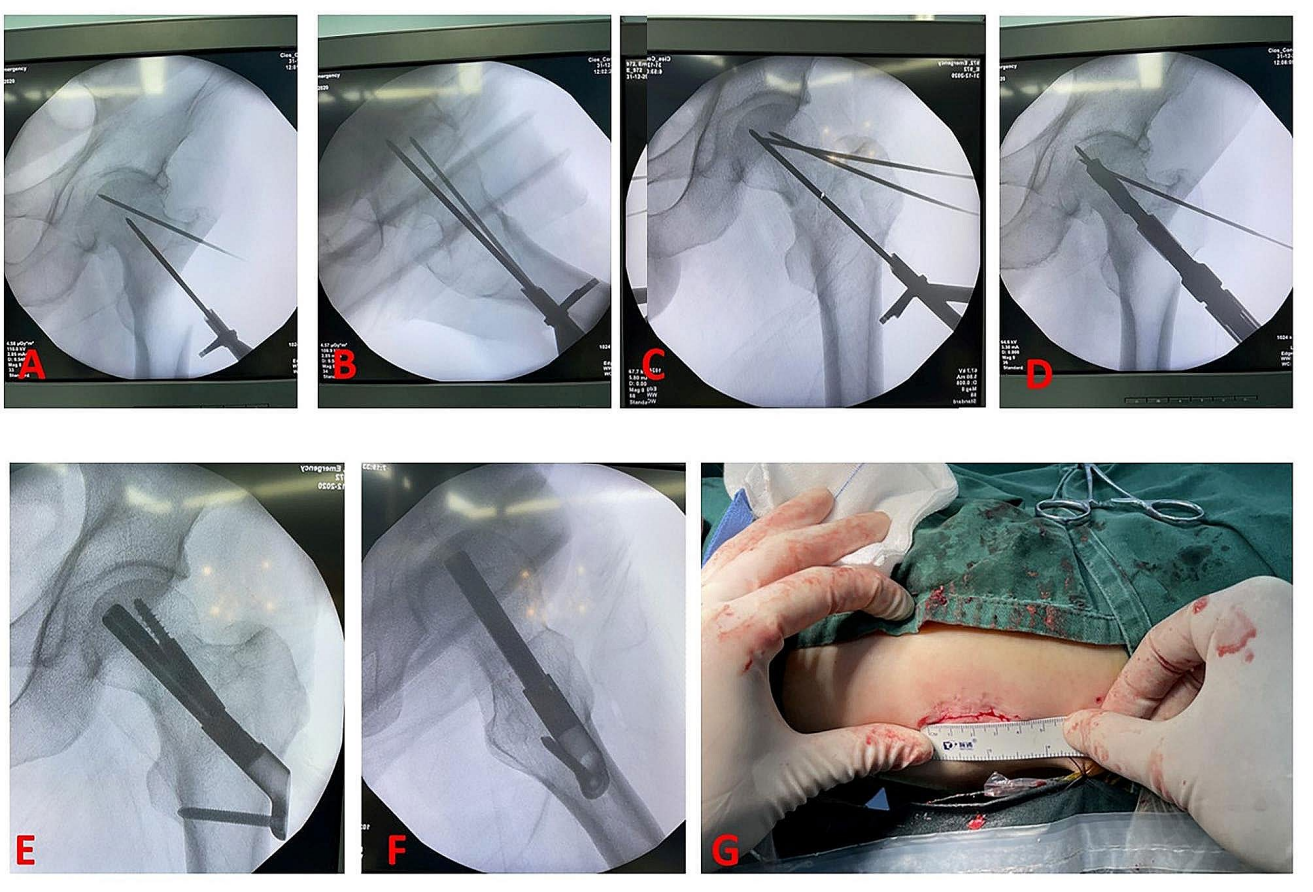
Surgical technique of FNS. (**A**) and (**B**), Insert an anti-rotation wire and guide wire. (**C**), reduction and temporary fixation. (**D**), ream is until 5 mm away from the subchondral bone. E and F, insert the FNS. G, Incision length

A schematic diagram of the FNS procedure is shown in Fig. 2. Closed reduction was performed under fluoroscopy. After the confirmation of good reduction quality (evaluated by the Garden alignment index), the FNS were inserted for internal fixation. If the reduction is insufficient consider open reduction. Then, insert an unused wire as an antirotation wire in the superior/anterior part of the femoral neck to prevent any inadvertent rotation of the femoral head. Make a straight lateral skin incision of approximately 6 cm in length, starting 2 to 3 cm proximal to the center of the femoral neck axis. Ensure adequate exposure of the lateral femoral surface for proper hardware placement. Insert a guide wire in both anterior positions and lateral positions, ensuring they are positioned at the center of the femoral neck, with the tip of the guide pin positioned 5 mm away from the subchondral bone. Measure the length and carefully ream dense bone, avoiding excessive reaming. Insert the implant over the central guide wire into the pre-reamed hole manually. Use image intensification to confirm the insertion depth and ensure that the plate is inserted down to the bone as well as aligned with the axis of the femoral shaft. Insert the anti-rotation screw with the selected construct size. Insert the locking screw with the determined length, as read from the drill bit or depth gauge. After confirming satisfactory fracture reduction and internal fixation position through fluoroscopy, remove the anti-rotation wire, and suture the wound.

### Postoperative managements

**Fig. 3:**
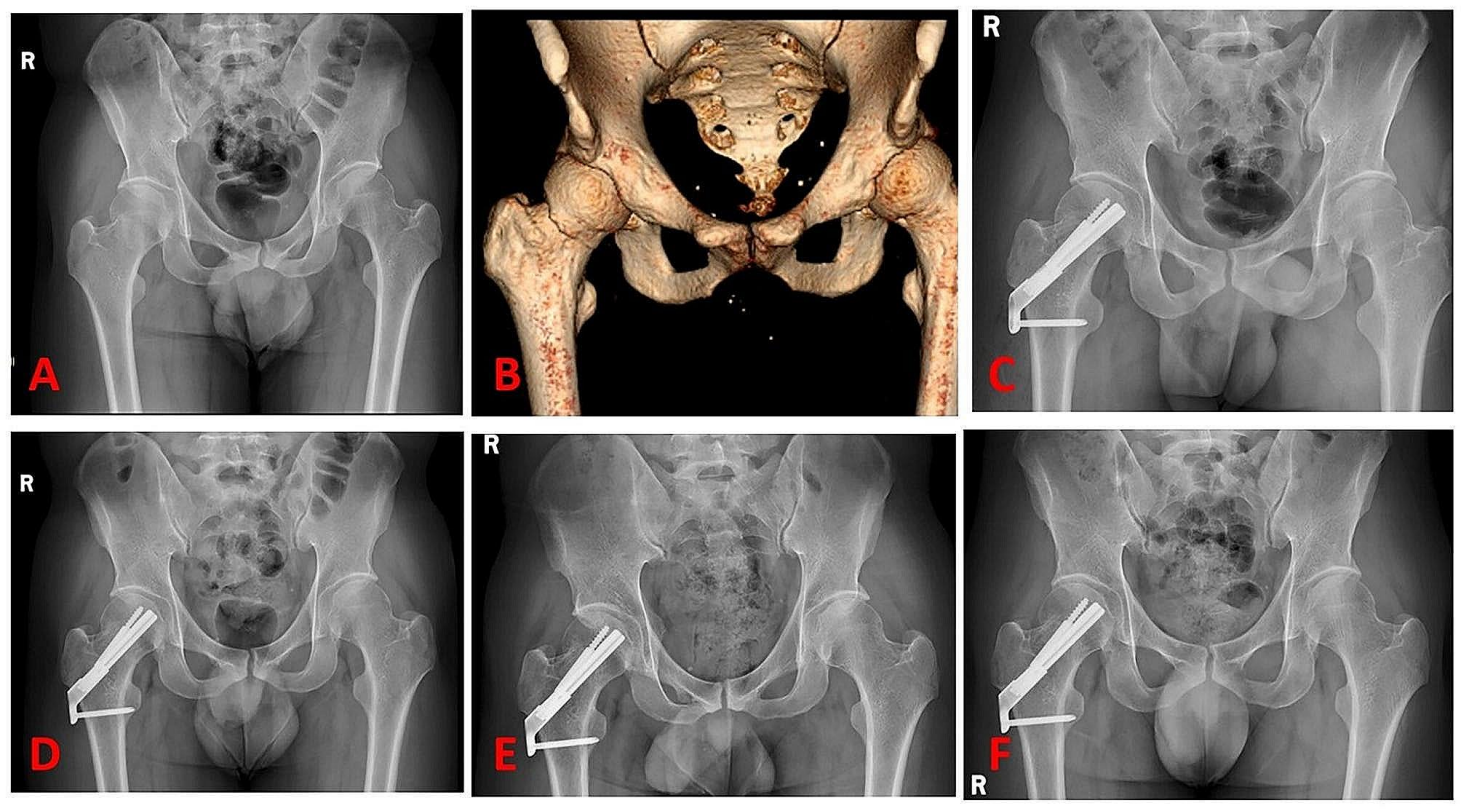
A 45-year-old male patient suffered right femoral neck fracture. (**A**) and (**B**) showed the femoral neck fracture. (**C**), x-ray postoperative. (**D**), X-ray three months after operation. (**E**), six months after operation. (**F**), one year after operation

In addition to regular wound checks, there are no restrictions on patient positioning. Patients were encouraged to perform non-weight bearing exercises when pain could be tolerated. Partial weight bearing was allowed at that time and progressed gradually according to the condition of each patient’s associated injuries. All patients underwent follow-up appointments at 6 weeks, 3 months, 6 months, and 12 months postoperatively (Fig. 3).

### Outcome evaluation

All the patients had AP and LP fluoroscopies of the hip intraoperatively and postoperatively. The radiographs were analyzed and measured through the Picture Archiving and Communication System (PACS). The length of femoral neck shortening (LFNS) was measured on the anteroposterior radiograph by comparing the position of the implant between the immediate postoperative film and the latest follow-up radiograph. This pragmatic approach, utilized in other studies measuring femoral neck shortening, provides a practical measure. The functional status at the last follow-up was assessed using the Harris functional scoring criteria [15]. Total score is 100, ≥ 90 is excellent, 80–89 is good, 70–79 is fair, and less than 70 is poor.

We also tracked patients for the following complications: avascular necrosis of the femoral head, nonunion, malreduction, implant failure (internal fixation screw breakage, implant cut-out), wound infection, heterotopic ossification (HO), need for revision surgery, delayed ambulation. We defined nonunion as either a loss of reduction or fixation observed after six weeks or as radiological evidence of the absence of union at one year, in accordance with definitions used in previous studies [16, 17].

### Statistical analysis

Data were analyzed using one-way analysis of variance and the chisquared test. *P* < 0.05 was considered statistically significant. Data are given as mean ± standard deviation. Statistical analyses were performed using SPSS version 20.0 software.

## Results

### Patient characteristics

A total of 120 patients with a mean age of 40.4 years (ranging from 21 to 58 years), were included in the final analysis. The demographics, cause of injury, smoking, time to surgery, Garden fracture classification (non-displaced or displaced), Pauwels classification and ASA are shown in Table 1. No significant differences were found between the three groups.

**Table 1:** Patients characteristics

Variable	FNS(*n* = 40)	MCS(*n* = 40)	DHS(*n* = 40)	F or X^2^	P	
Age(year)	41.5 ± 9.27	40.9 ± 10.97	38.7 ± 11.12	F = 0.79	*P* = 0.456	
Gender				X^2^ = 1.49	*P* = 0.476	
Male	29(72.5%)	30(75%)	31(77.5%)			
Female	11(27.5%)	10(25%)	9(22.5%)			
BMI	24.89 ± 1.87	24.19 ± 1.76	24.57 ± 2.05	F = 1.37	*P* = 0.259	
Cause of injury				X^2^ = 1.913	*P* = 0.928	
Falling from heights	11(27.5%)	9(22.5%)	12(30%)			
Slip and fall	4(10%)	3(7.5%)	5(12.5%)			
Traffic accident	20(50%)	21(52.5%)	19(47.5%)			
Other reason	5(12.5%)	7(17.5%)	4(10%)			
Side of injured				X^2^ = 0.94	*P* = 0.626	
Right	17(42.5%)	20(50%)	15(37.5%)			
Left	23(57.5%)	20(50%)	25(62.5%)			
Smoking				X^2^ = 1.182	*P* = 0.554	
Yes	28	31	32			
No	12	9	8			
Garden Classification				X^2^ = 1.28	*P* = 0.526	
Non-displaced	25(62.5%)	22(55%)	20(50%)			
Displaced	15(37.5%)	18(45%)	20(50%)			
Pauwels Classification				X^2^ = 0.64	*P* = 0.959	
I	4(10%)	5(12.5%)	6(15%)			
II	21(52.5%)	22(55%)	20(50%)			
III	15(37.5%)	13(32.5%)	14(35%)			
Time from injury to surgery(day)	2.15 ± 0.70	1.9 ± 0.74	2.02 ± 0.72	F = 1.22	*P* = 0.299	
ASA score	1.53 ± 0.55	1.58 ± 0.59	1.45 ± 0.55	F = 0.49	*P* = 0.613	

### Perioperative data

For the perioperative data was showed in Table 2, DHS group showed longer operation time(52.15 ± 4.80 min), more blood loss(59.05 ± 5.87 ml) and longer time of hospitalization(7.6 ± 0.90 d) than FNS group (39.65 ± 2.84 min, 45.33 ± 9.63 ml and 4.87 ± 0.48 d) and MCS group (39.45 ± 3.10 min, 48.15 ± 7.88 ml and 5.04 ± 0.49 d) (*p* < 0.05). In addition, the time of fluoroscopy in FNS group (15.45 ± 3.67) was less than that in MCS group (26.3 ± 4.76) and DHS group (27.1 ± 5.67) (*p* < 0.05). what’s more, the cost of FNS group(44.51 ± 2.99 thousand RMB) is significantly higher than that of group MCS(15.68 ± 3.89 thousand RMB) and DHS(16.34 ± 3.99 thousand RMB)(*p* < 0.05).

**Table 2:** Comparison of perioperative data between the two groups

Variable	FNS(*n* = 40)	MCS(*n* = 40)	DHS(*n* = 40)	F or X^2^	P
Operation time(min)	39.65 ± 2.84	39.45 ± 3.10	52.15 ± 4.80	F = 155.8	*P* = 0.001*
Blood loss(ml)	45.33 ± 9.63	48.15 ± 7.88	59.05 ± 5.87	F = 33.33	*P* = 0.001*
Time of fluoroscopy	15.45 ± 3.67	26.3 ± 4.76	27.1 ± 5.67	F = 74.51	*P* = 0.001*
Time of hospitalization(day)	4.87 ± 0.48	5.04 ± 0.49	7.6 ± 0.90	F = 219.73	*P* = 0.001*
Cost of hospitalization(RMB, thousand)	44.51 ± 2.99	15.68 ± 3.89	16.34 ± 3.99	F = 811.61	*P* = 0.001*

### Clinical and radiological outcomes

**Fig. 4:**
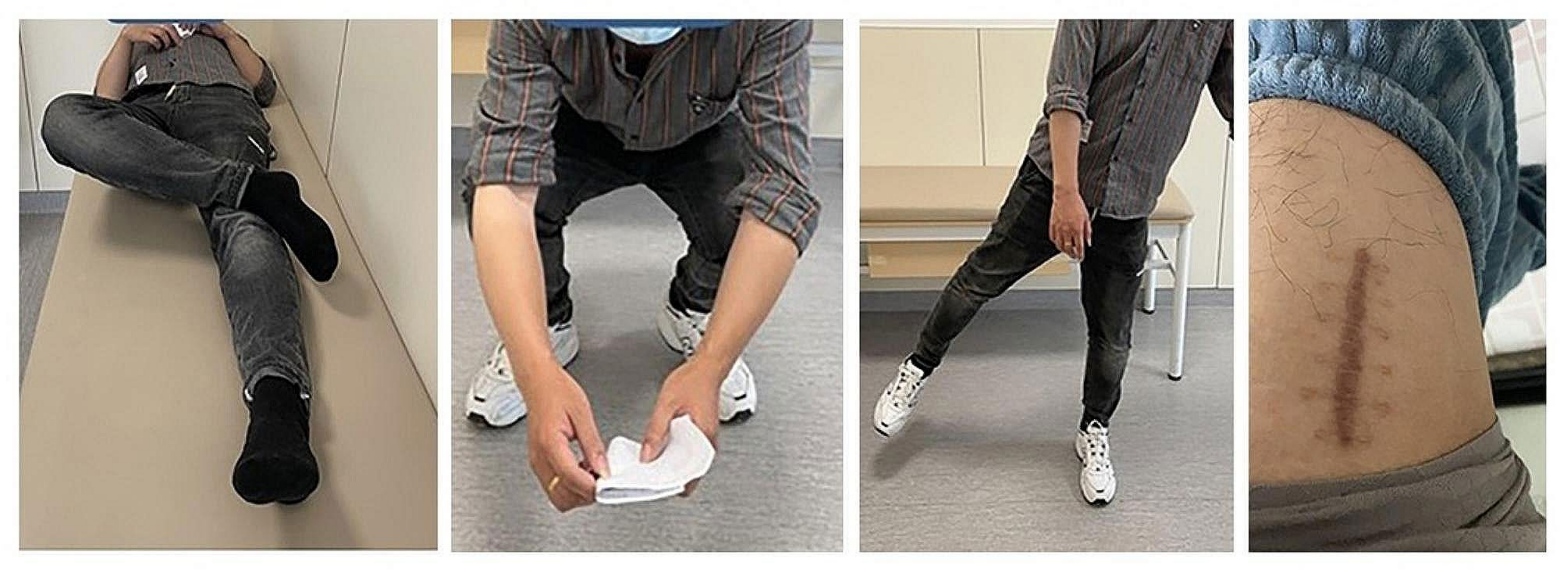
Functional follow-up and incision in patients with femoral neck fracture one year after operation

The clinical and radiological outcomes were showed in Table 3. All patients were followed for more than 12 months, there was no significant difference. The FNS, MCS and DHS groups showed a similar mean LFNS of 4.1 ± 1.12 mm, 3.74 ± 1.22 mm, and 3.85 ± 1.18 mm, respectively. Case of femoral neck shortening > 5 mm in FNS(9,22.5%), MCS(9,22.5%) and DHS(8,20%) groups were also the same. The two radiographic analysis showed there was no significant difference in the three groups. Via follow-up, we found the hip function of the patients in three groups was improved (Fig. 4). The mean Harris score of FNS group was 91.78 ± 3.71, whereas that of MCS group was 91.03 ± 4.2, DHS group was 91.36 ± 3.83. No significant difference was noted in Harris score between the three groups (*P* = 0.70).

**Table 3:** Clinical and radiological parameters at the latest follow-up

Variable	FNS(*n* = 40)	MCS(*n* = 40)	DHS(*n* = 40)	F or X^2^	P
Follow-up duration (month)	13.8 ± 0.88	13.8 ± 0.76	13.45 ± 0.88	F = 2.31	*P* = 0.104
Length of femoral neck shortening(mm)	4.1 ± 1.12	3.74 ± 1.22	3.85 ± 1.18	F = 0.99	*P* = 0.372
Femoral neck shortening > 5 mm	9(22.5%)	9(22.5%)	8(20%)	X^2^ = 0.098	*P* = 0.952
Harris score	91.78 ± 3.71	91.03 ± 4.20	91.36 ± 3.83	F = 0.365	*P* = 0.70

### Complications

**Fig. 5:**
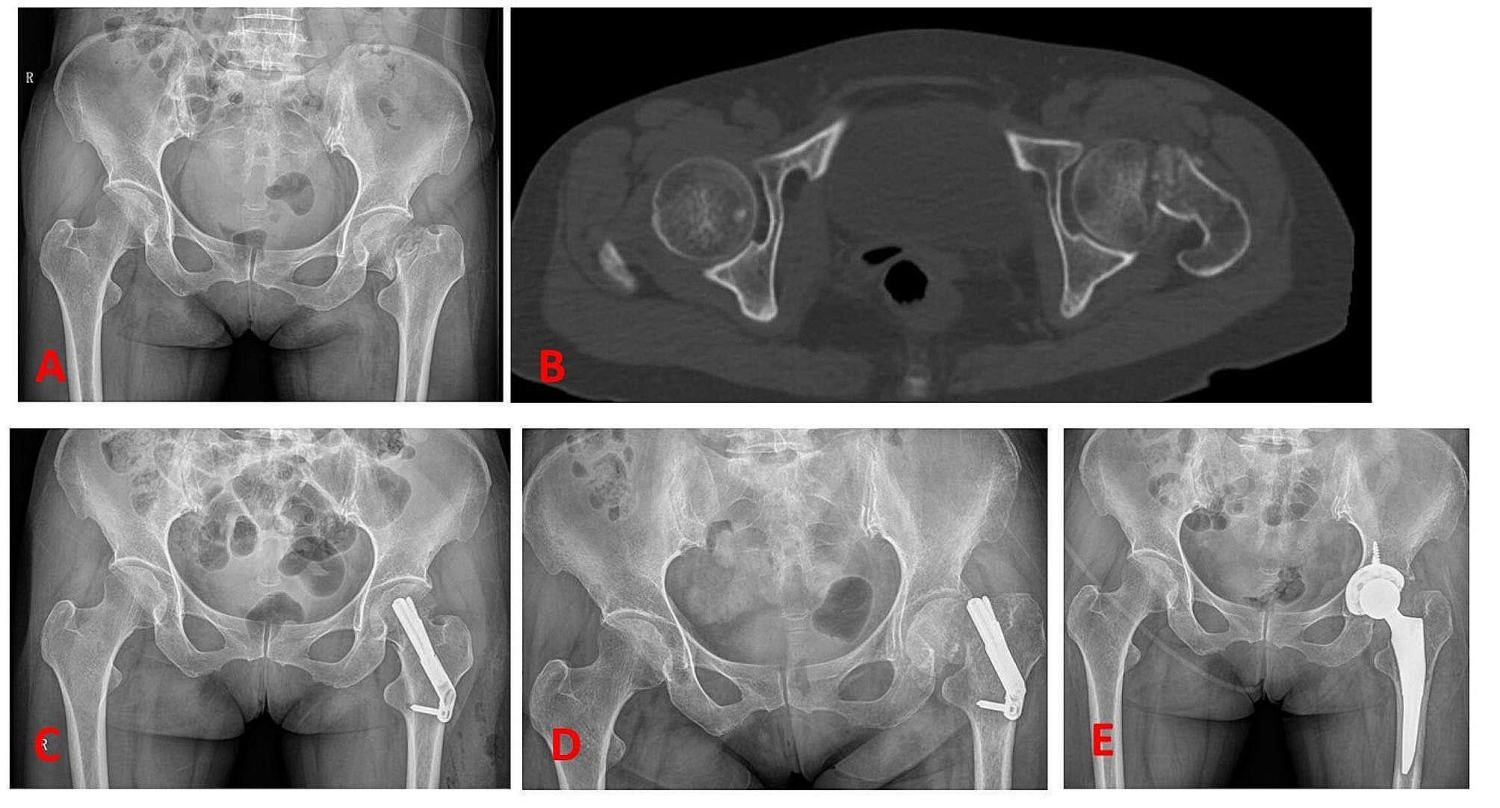
A 55-year-old female patient suffered left femoral neck fracture in the FNS group. (**A**) and (**B**) showed the femoral neck fracture. (**C**), X-ray postoperative. (**D**), x-ray with Screw cutting six months after operation. (**E**), X-ray with total hip replacement

The complications of three groups were summarized in Table 4. Superficial wound infection was present in one case in each group, and resolved with wound cleansing and antibiotics. There was no deep infection and other wound-healing complications. There were 2 patients in FNS group (Figs. 5), 2 patients in MCS group and 3 patients in DHS group suffered internal fixation failure. The FNS, MCS and DHS groups showed a similar mean rate AVN of 4(10%), 4(10%) and 5(12.5%), respectively(*P* > 0.05). All patients with internal fixation failure and AVN underwent total hip replacement and recovered well after surgery.

**Table 4:** Comparison of complications between the two groups

Complications	FNS(*n* = 40)	MCS(*n* = 40)	DHS(*n* = 40)	X^2^	P
Wound superficial infection	1(2.5%)	1(2.5%)	1(2.5%)		*P* = 1
Internal fixation failure	2(5%)	2(5%)	3(7.5%)	X^2^ = 0.303	*P* = 0.859
Avascular necrosis of femoral head	4(10%)	4(10%)	5(12.5%)	X^2^ = 0.174	*P* = 0.917
Total	7(17.5%)	7(17.5%)	9(22.5)	X^2^ = 0.43	*P* = 0.806

## Discussion

Femoral neck fracture is one of the common fractures of lower limbs. Surgical treatment of femoral neck fractures mainly involves internal fixation and arthroplasty. However, arthroplasty may not be a good choice for young patients due to their higher fracture healing potential, bone density, and life expectancy compared to elderly patients. Currently commonly used internal fixation options for young patient include: 3 or 4 parallel cannulated compression screws [6]; DHS or combined with an additional hollow screw [18]; three cannulated screws combined with inner steel plate or “F type” screws [19].

MCS and DHS are the most common implants for internal fixation of femoral neck fractures. While MCS offers a minimally invasive approach, it may exhibit lower biomechanical stability when compared to DHS. Although DHS is more biomechanically stable, larger surgical trauma is inevitable. FNS is specifically designed to address femoral neck fractures, offering enhanced stability and aiming to reduce the need for re-operations related to fixation complications [20]. It is characterized by providing angular stability combined with a minimally invasive surgical technique and the opportunity of additional intraoperative compression at the fracture site if needed. Schopper C [21] evaluated the biomechanical performance of the FNS versus the Hansson Pin System (Hansson Pins) with two parallel pins in a Pauwels II femoral neck fracture model with posterior comminution. They found that the FNS can provide superior resistance against varus deformation and perform in a less sensitive way to variations in implant placement. Stoffel K, et al. [10] evaluated the biomechanical performance FNS in comparison with DHS and MCS for fixation of femoral neck fractures in a cadaveric model. At last, from a biomechanical point of view, the FNS is a valid alternative to treat unstable femoral neck fractures, representing the advantages of a minimally invasive implant with comparable stability to the 2 DHS systems and superior to cannulated screws [22]. This study compared the clinical data of 120 patients with fresh femoral neck fractures treated with FNS, MCS, and DHS. The study aimed to explore and analyze the differences in the effectiveness of these three internal fixation methods in actual clinical applications. The results showed that FNS had several advantages, including shorter operation time, reduced blood loss, decreased fluoroscopy time, and shorter hospitalization duration. However, it was noted that the hospitalization cost associated with FNS was higher.

Hip fractures treated with osteosynthesis have a risk of femoral neck shortening which can result in a difference of leg length. The degree of femoral neck shortening was categorized into mild (shortening ≤ 5 mm), moderate (shortening 5–10 mm), and severe (shortening > 10 mm), with a more severe degree indicating a worse prognosis of hip joint function. The FAITH trial showed comparable femoral neck shortening of DHS compared to CCS [8].

In our study, patients with femoral neck shortening > 5 mm accounted for 22.5% (9/40), 22.5% (9/40), and 20% (8/40) in the FNS, MCS, and DHS groups, respectively. At the latest follow-up, no significant difference was found in LFNS and the proportion of femoral neck shortening (> 5 mm) between the three groups. Compared to the study the rate of femoral neck compression over 5 mm of the FNS in our study was comparable. However Kai Wang, et al. [23] reported the incidence of femoral neck shortening after femoral neck fixation was 39.1%, and this may be related to the large proportion of Garden III and IV types (51/87). In this study, the rate of Garden III and IV types was 44.1% (53/120). In this study, the Harris scores at the last follow-up were similar among the three groups which indicated the reliability of FNS fixation.

The most serious complication of femoral neck fracture is avascular necrosis of the femoral head and internal fixation failure [24, 25]. Several factors, such as the patient’s age, overall health status, comorbidities, injury mechanism, fracture displacement, and osteoporosis, can contribute to these complications, and these factors are often beyond the control of healthcare providers [26, 27]. There were some complications in the three groups, but no special differences, which was consistent with current literature.

Meanwhile, from this series of cases, we have some valuable experience with FNS. Firstly, when insert the antirotation wire, we should reserve a channel for FNS. The Kirschner wire is generally fixed in the upper 1/3 of the femoral head in the AP position, while avoiding the center of the femoral neck in the lateral position. Secondly, the placement of FNS is percussion to avoid violence and rotation, and the forward tilt angle needs to be adjusted. It avoids the secondary rotational movement of the fracture caused by the rotational torsion. Use the connecting rod to insert, the tip of the power rod is recommended to be 5–10 mm below the cartilage of the femoral head. Thirdly, there is a compression space of 20 mm for FNS, which is conducive to fracture healing. In addition, due to the sliding mechanism during the fracture healing process, the absorption of the fractured end of the femoral neck can realize the re-contact of the fracture.

This retrospective cohort study had certain limitations. It may be subject to selection bias, and the relatively small sample size might lack the statistical power needed to detect subtle differences. Therefore, the results of this study warrant validation through a multicenter, large-sample, prospective randomized controlled trial. Second, this study was a comparative study with a short follow-up period. Consequently, it was unable to assess long-term outcomes, such as the occurrence of avascular necrosis of the femoral head. Future studies should consider extending the follow-up duration for a more comprehensive assessment.

## Conclusion

In summary, FNS, MCS, and DHS are effective for the young femoral neck fractures. The study revealed no significant differences in complication rates among the three groups. However, a lower time of fluoroscopy of the FNS shortens the operation time. Minimally invasive procedures are associated with less blood loss and less hospital stay. One notable drawback is its higher cost.

## Data Availability

We do not wish to share our data, because some of patients’ data regarding individual privacy, and according to the policy of our hospital, the data could not be shared to others without permission. However, the data are available from the corresponding author on reasonable request.
